# Supplementary material for: Dicer1 Depletion Leads to DNA Damage Accumulation and Cell Death in a RET/PTC3 Papillary Thyroid Cancer Mouse Model, Thereby Inhibiting Tumor Progression
Source: Cells. 2025 Sep 19;14(18):1465. doi: 10.3390/cells14181465 (PMC12468101; doi:10.3390/cells14181465)
Supplement: Supplementary file 1 [file cells-14-01465-s001.zip › cells-3845397-supplementary.pdf]

SUPPLEMENTARY

	FORWARD	REVERSE
Bax	5´CGGCGAATTGGAGATGAACT3´	5´GCAAAGTAGAAGAGGGCAA3´
Bcl2	5´TGACTTCTCTCGTCGCTACC3´	5´GGTGACATCTCCCTGTTGAC3´
Bim	5´CGTCCACCCAATGTCTGACT3´	5´GCTGCAATTCTCCACCTTCT3´
Dicer1	5'AGCTTGAGAAGAACGAAATGC3´	5´TCATCGGATAGTACACCTGCC3´
Gpx2	5´CAATGAGCTGCAATGTCGCTT3´	5´TCGTTCTGACAGTTCTCCTGA3´
Hprt	5´GCTACTGTAATGATCAGTCAACGGG3´	5´AAGCTTGCAACCTTAACCATTTTG3´
Nis	5´AGCTGCCAACACTTCCAGAG3´	5´GATGAGAGCACCACAAAGCA3´
Nkx2.1	5´GGCGCCATGTCTTGTTCT3´	5´GGGCTCAAGCGCATCTCA3´
Nqo1	5´AGGATGGGAGGTACTCGAATC3´	5´AGGCGTCCTTCCTTATATGCTA3´
Ogg1	5´CCTAGCAGCATGAGACATCGC3´	5´CAGTGTCCATACTTGATCTGCC3´
Pax8	5´CAGCCTGCTGAGTTCTCCAT3´	5´CTGTCTCAGGCCAAGTCCTC3´
Tbp	5´TGTACCGCAGCTTCAAAATATTGTAT3´	5´AAATCAACGCAGTTGTCCGTG3´
Tg	5´GTCCAATGCCAAAATGATGGTC3´	5´GAGAGCATCGGTGCTGTTAAT3´
Tpo	5´ACAGTCACAGTTCTCCACGGATG3´	5´ATCTCTATTGTTGCACGCCCC3´
Tshr	5´GTCTGCCCAATATTTCCAGGATCTA3´	5´GCTCTGTCAAGGCATCAGGGT3´

Table S1. Sequence of the primers used for RT-qPCR experiments.

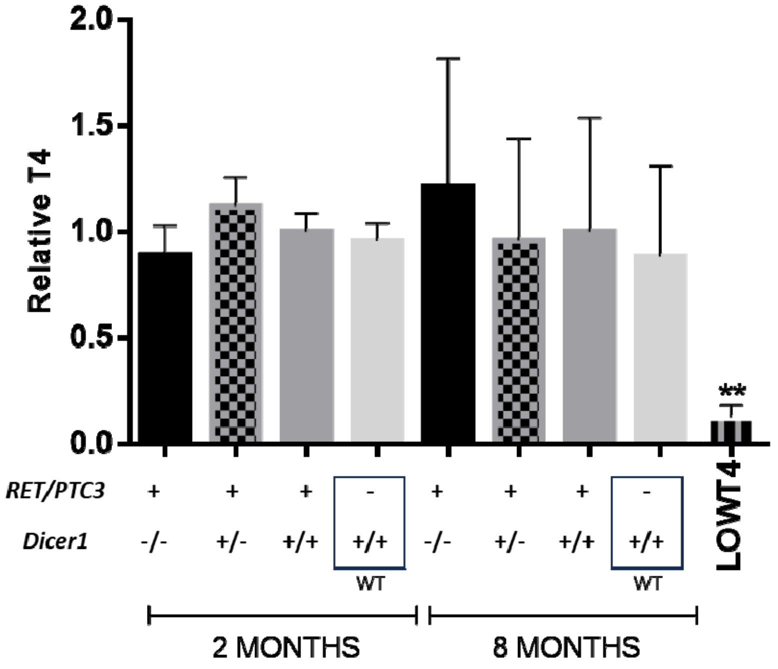

Supplementary Figure S1. Normal T4 serum levels are maintained in thyroid tumors from RET/PTC3 *Dicer1*$^{(-/-)}$, RET/PTC3 *Dicer1*$^{(+/-)}$ and RET/PTC3 *Dicer1*$^{(+/+)}$ mice. Serum levels of T4 were assessed by ELISA in 2-month-old (RET/PTC3 *Dicer1*$^{(-/-)}$ n=6, RET/PTC3 *Dicer1*$^{(+/-)}$ n=5, RET/PTC3 *Dicer1*$^{(+/+)}$ n=4, WT n=7) or 8-month-old (RET/PTC3 *Dicer1*$^{(-/-)}$ n=13, RET/PTC3 *Dicer1*$^{(+/-)}$ n=15, RET/PTC3 *Dicer1*$^{(+/+)}$ n=28, WT n=6) mice as well as in mice with hypothyroidism (LOWT4, n=5) and normalized by the mean of the RET/PTC3*+ Dicer1*$^{(+/+)}$ control group. Statistically significant differences were determined using Kruskal–Wallis test ** $p < 0.01$. The mean of each column was compared to the mean of the WT column. The columns represent the mean values, and the error bars indicate the standard deviation (mean ± SD).

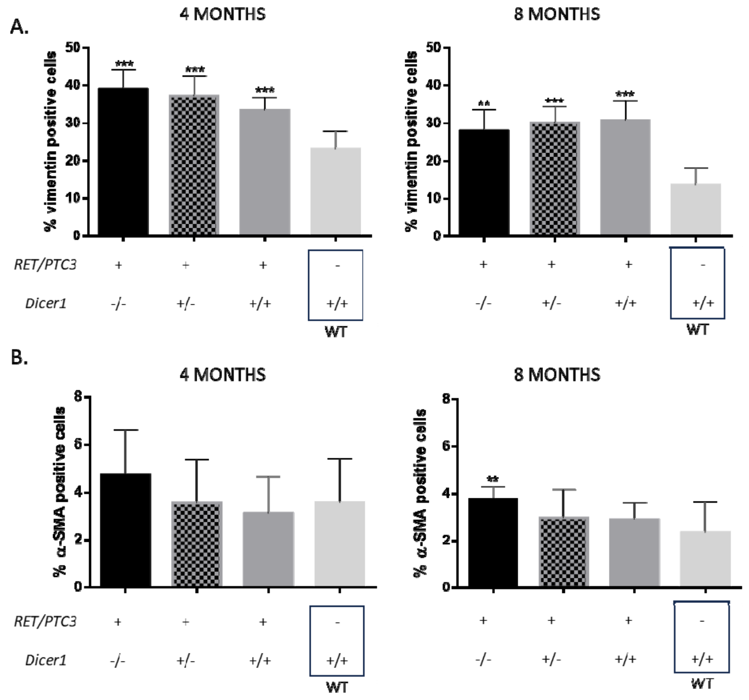

Supplementary Figure S2. Thyroid tumors from 4- and 8-month-old mice exhibit increased number of vimentin-positive cells without changes in the percentages of α-SMA-positive cells. (A) Quantification of the percentage of vimentin-positive cells (number of vimentin-positive cells/number of DAPI stained cells) (B) Quantification of the percentage of α-SMA-positive cells (number of α-SMA-positive cells/number of DAPI stained cells) in thyroids from 4-month-old (RET/PTC3 *Dicer1*$^{(-/-)}$ n=7, RET/PTC3 *Dicer1*$^{(+/-)}$ n=6, RET/PTC3 *Dicer1*$^{(+/+)}$ n=8, WT n=6) or 8-month-old (RET/PTC3 *Dicer1*$^{(-/-)}$ n=12, RET/PTC3 *Dicer1*$^{(+/-)}$ n=12, RET/PTC3 *Dicer1*$^{(+/+)}$ n=14, WT n=10) mice. Statistically significant differences were determined using ANOVA test. ** $p < 0.01$ and *** $p < 0.001$. The mean of each column was compared to the mean of the WT column. The columns represent the mean values, and the error bars indicate the standard deviation (mean ± SD).

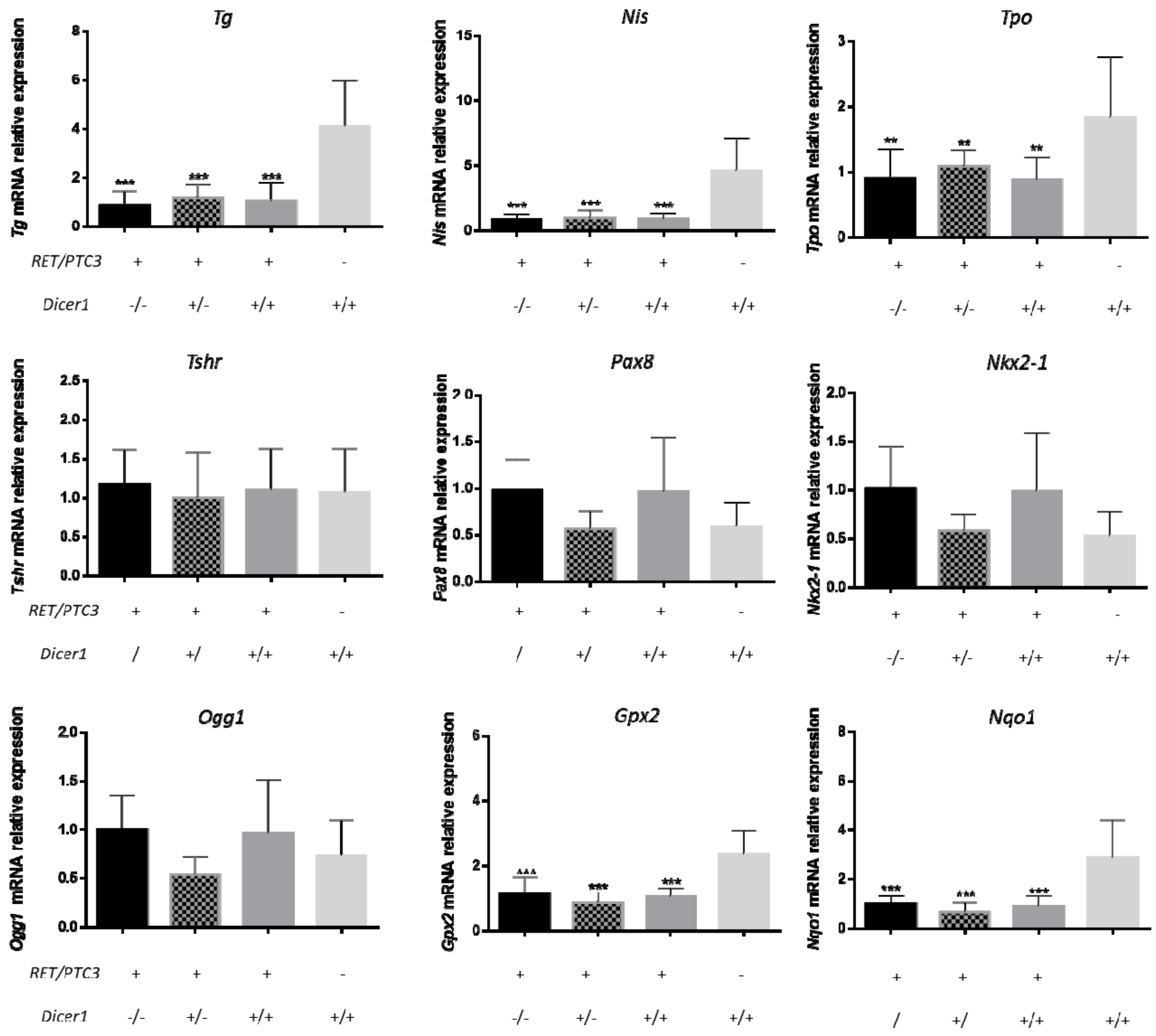

Supplementary Figure S3. Altered expression of thyroid differentiation and redox genes in RET/PTC3-induced thyroid tumors from 8-month-old mice is not influenced by partial or total loss of *Dicer1*. RT-qPCR analysis of mRNA expression of *thyroglobulin* (*Tg*), *Nis* (Na(+)/I(-) symporter), *thyroid peroxidase* (*Tpo*), *thyroid stimulating hormone receptor* (*Tshr*), *Paired box 8* (*Pax8*), *NK2 Homeobox 1* (*Nkx2-1), 8-Oxoguanine DNA Glycosylase* (*Ogg1*), *Glutathione Peroxidase 2* (*Gpx2*) and *NAD(P)H Quinone Dehydrogenase 1* (*Nqo1*) in thyroids of 8-month-old mice (RET/PTC3 *Dicer1*$^{(-/-)}$ n=8, RET/PTC3 *Dicer1*$^{(+/-)}$ n=10, RET/PTC3 *Dicer1*$^{(+/+)}$ n=10, WT n=8). Statistically significant differences were determined using ANOVA test. ** p < 0.01 and *** p < 0.001. The mean of each column was compared to the mean of the WT column. The columns represent the mean values, and the error bars indicate the standard deviation (mean ± SD).

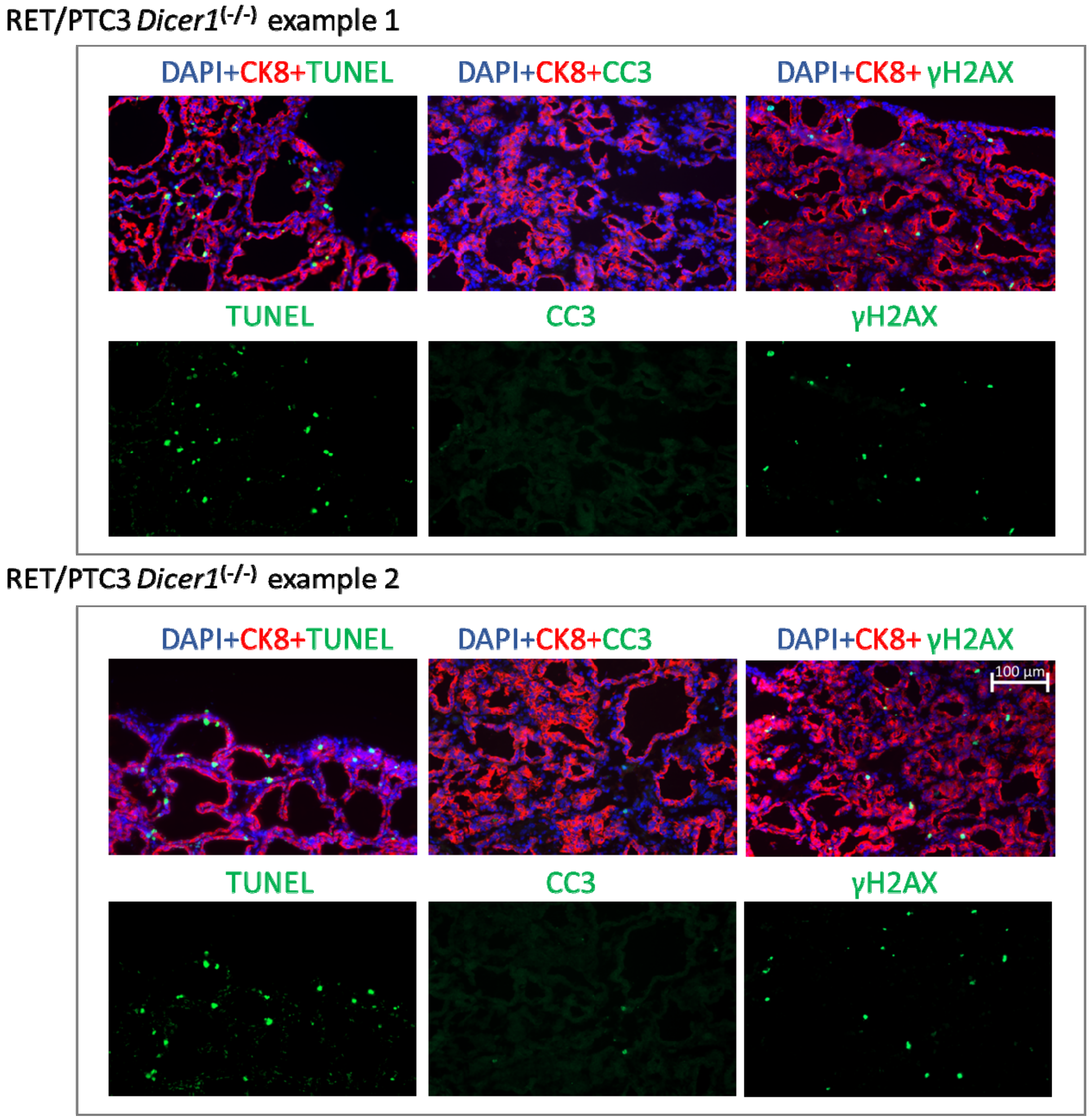

Supplementary Figure S4. TUNEL and γH2AX staining reveal DNA damage–induced, cleaved caspase-3–independent, cell death in RET/PTC3 *Dicer1*$^{(-/-)}$ thyroid tumors. Representative images from two representative RET/PTC3 *Dicer1*$^{(-/-)}$ thyroid tumors stained for DAPI and immunolabelled against cytokeratin-8 (CK8, red) and TUNEL, cleaved-caspase-3 (CC3) or γH2AX (green) (OCT slides). Images were captured at 20x magnification. Scale bar 100µm.
